# A randomised controlled trial evaluating arrhythmia burden, risk of sudden cardiac death and stroke in patients with Fabry disease: the role of implantable loop recorders (RaILRoAD) compared with current standard practice

**DOI:** 10.1186/s13063-019-3425-1

**Published:** 2019-05-31

**Authors:** Ravi Vijapurapu, Rebecca Kozor, Derralynn A. Hughes, Peter Woolfson, Ana Jovanovic, Patrick Deegan, Rosemary Rusk, Gemma A. Figtree, Michel Tchan, David Whalley, Dipak Kotecha, Francisco Leyva, James Moon, Tarekegn Geberhiwot, Richard P. Steeds

**Affiliations:** 1 Department of Cardiology, Queen Elizabeth Hospital Birmingham, Mindlesohn Way, Birmingham, B15 2TH UK; 2 Institute of Cardiovascular Sciences, University of Birmingham, Edgbaston, Birmingham, B15 2TT UK; 3 Department of Endocrinology, Queen Elizabeth Hospital Birmingham, Mindlesohn Way, Birmingham, B15 2TH UK; 4 Sydney Medical School, University of Sydney, Sydney, 2006 NSW Australia; 5 Cardiology Department, Royal North Shore Hospital, Reserve Road, St. Leonards, NSW 2065 Australia; 6 Lysosomal Storage Disorder Unit, Royal London NHS Foundation Trust, University College London, Pond Street, London, NW3 2QG UK; 7 Department of Cardiology, Salford Royal Hospital, Stott Lane, Salford, M6 8HD UK; 8 Mark Holland Metabolic Unit, Salford Royal Hospital, Stott Lane, Salford, M6 8HD UK; 9 Department of Medicine, Addenbrooke’s Hospital, Hill Road, Cambridge, CB2 0QQ UK; 10 Department of Cardiology, Addenbrookes Hospital, Hill Road, Cambridge, CB2 0QQ UK; 11 Department of Genetics, Westmead Hospital, Hawkesbury Road, Westmead, NSW 2145 Australia; 12 Aston Medical Research Institute, Aston Medical School, Birmingham, B4 7ET UK; 13 Barts Heart Centre, Barts Health NHS Trust, 16-18 Westmoreland Street, London, W1G 8PH UK; 14 Institute of Metabolism and System Research, University of Birmingham, Edgbaston, Birmingham, B15 2TT UK

**Keywords:** Fabry, Arrhythmia, ILR, Cardiomyopathy

## Abstract

**Background:**

Fabry disease (FD) is a genetic disorder caused by a deficiency in the enzyme alpha-galactosidase A, leading to an accumulation of glycosphingolipids in tissues across the body. Cardiac disease is the leading cause of morbidity and mortality. Advanced disease, characterised by extensive left ventricular hypertrophy, ventricular dysfunction and fibrosis, is known to be associated with an increase in arrhythmia. Data identifying risk factors for arrhythmia are limited, and no Fabry-specific risk stratification tool is available to select those who may benefit from initiation of medical or device therapy (implantable cardiac defibrillators). Current monitoring strategies have a limited diagnostic yield, and implantable loop recorders (ILRs) have the potential to change treatment and clinical outcomes.

**Aim:**

The aim of this study is to determine whether ILRs can (1) improve arrhythmia detection in FD and (2) identify risk predictors of arrhythmia.

**Methods:**

A prospective, 5-year, open-label, international, multi-centre randomised controlled trial of a minimum of 164 participants with genetically or enzymatically confirmed FD (or both) who have evidence of cardiac disease will be recruited from five centres: Queen Elizabeth Hospital, Birmingham, UK; Salford Royal Hospital, Salford, UK; Royal Free Hospital, London, UK; Addenbrookes Hospital, Cambridge, UK; and Westmead Hospital, Sydney, Australia. Participants will be block-randomised (1:1) to two study arms for cardiac monitoring (i) control arm: standard of care with annual 24 h or 5-day Holter monitor or (ii) treatment arm: continuous cardiac monitoring with ILR implantation plus standard of care. Participants will undergo multiple investigations—blood/urine biomarkers, 12-lead and advanced electrocardiogram (ECG) recording, echocardiography and cardiovascular magnetic resonance (CMR) imaging—at baseline and 6–12 monthly follow-up visits. The primary endpoint is identification of arrhythmia requiring initiation or alteration in therapy. Secondary outcome measures include characterising the risk factors associated with arrhythmia and outcome data in the form of imaging, ECG and blood biomarkers.

**Discussion:**

This is the first study evaluating arrhythmia burden and the use of ILR across the spectrum of risk profiles in Fabry cardiomyopathy. This will enable detailed characterisation of arrhythmic risk predictors in FD and ultimately support formulation of Fabry-specific guidance in this high-risk population.

**Trial registration:**

ClinicalTrials.gov (NCT03305250). Registered on 9 October 2017.

**Electronic supplementary material:**

The online version of this article (10.1186/s13063-019-3425-1) contains supplementary material, which is available to authorized users.

## Background

Fabry disease (FD) is an X-linked lysosomal storage disorder in which a deficiency in the enzyme alpha-galactosidase A [1] leads to a progressive accumulation of glycosphingolipids such as globotriaosylceramide (Gb3) and globotriaosylsphingosine (lyso-Gb3) in tissues across the body [2]. Cellular changes within the tissue microenvironment lead to extensive cardiovascular, neurological and renal dysfunction. Clinical presentation can be extremely variable [3] and developments in therapy have changed the natural progression of FD, such that cardiac disease has surpassed renal involvement as the leading cause of morbidity and mortality [4]. Cardiovascular involvement is characterised by progressive glycosphingolipid storage, inflammation, left ventricular hypertrophy (LVH), fibrosis, arrhythmia, congestive cardiac failure and sudden death [5]. Although a significant proportion of cardiovascular deaths are ascribed to ‘sudden cardiac events’ and symptoms such as palpitations and syncope are almost universal, very little is known regarding the true frequency of arrhythmia. Registry data have suggested that the rate of atrial arrhythmia could be as high as 13% [6]. However, the incidence of ventricular arrhythmia varies widely (5% to 30%) [6]. Advanced cardiac disease characterised by extensive LVH, ventricular dysfunction and fibrosis is known to be associated with an increase in arrhythmia needing intervention. This includes bradyarrhythmia requiring a permanent pacemaker (PPM), ventricular arrhythmia requiring an implantable cardiac defibrillator (ICD) and atrial fibrillation (AF) requiring anticoagulation [7]. Limited data have identified LVH, the presence of late gadolinium enhancement (LGE) left atrial (LA) dilatation, a QRS duration of more than 120 ms and an elevated Mainz Severity Score Index (MSSI) as potential arrhythmic risk factors. However, there is no Fabry-specific risk calculator to select those who might benefit most from therapeutic interventions (such as ICDs) as there are for conditions such as hypertrophic cardiomyopathy (HCM) [8]. Novel electrical and blood biomarkers have also recently emerged as potential predictors of atrial and ventricular arrhythmia. Advances in technology have enabled computerised electrocardiogram (ECG) analysis using advanced signal averaging, vectorcardiographic reconstruction, singular value decomposition of QRS and T wave complexity, frequency content analysis, and beat-to-beat variability—collectively known as advanced ECG (A-ECG). These A-ECG measures can be applied retrospectively to digital files of clinically acquired ECGs, and outcome variables identified as potential arrhythmic risk markers [9–11]. These markers have a higher sensitivity and specificity than conventional ECG criteria in prediction of arrhythmic risk and assessment of conditions such as LVH and HCM [12, 13]. Cardiac involvement is a major determinant of morbidity and mortality in FD. ECG changes do not always reflect the extent of cardiac disease, making assessment with conventional ECG limited [14, 15]. Thus, detailed analysis of surface ECG markers through A-ECG could be a powerful tool not only to assess risk of arrhythmia but potentially to track cardiac pathology in FD.

Local myocardial inflammation has also been shown to alter electrophysiological properties and contribute to the arrhythmic vulnerability of cardiac tissue. Specific inflammatory biomarkers, such as interleukin-6 (IL-6) and high-sensitivity C-reactive protein (hs-CRP), have both been considered potential contributors to the pathophysiological mechanisms leading to the development of malignant ventricular arrhythmia and atrial arrhythmias, such as AF [16, 17]. Although there are no data on the role of IL-6 or hs-CRP in predicting arrhythmia in FD, the presence of scar identified by LGE on cardiovascular magnetic resonance (CMR) is known to be a risk factor and these lesions in some patients with FD have been linked to chronic inflammation [18]. As such, their evaluation may provide crucial information on the potential biochemical mechanisms underlying arrhythmia generation.

Current monitoring strategies for arrhythmia are limited to a 12-lead ECG at clinic visits, annual 24-h Holter monitoring, and response to a clinical event. However, a significant difficulty in clinical management is that, owing to the frequent nature of palpitations in patients with FD, symptomatic status loses specificity for identifying arrhythmia. Implantable loop recorders (ILRs) are small and subcutaneously inserted devices that enable continuous cardiac monitoring over the course of a 3-year period. These devices have the potential to modify care and outcomes by providing definite evidence of arrhythmia and to provide greater accuracy (due to length of recording) and earlier detection with remote/home monitoring, particularly in asymptomatic individuals. Although ILRs do not carry the same risk as other implanted devices, they are still invasive and have relatively high cost, so their use needs to be justified in the current healthcare climate.

## Methods

### Design

**Fig. 1:**
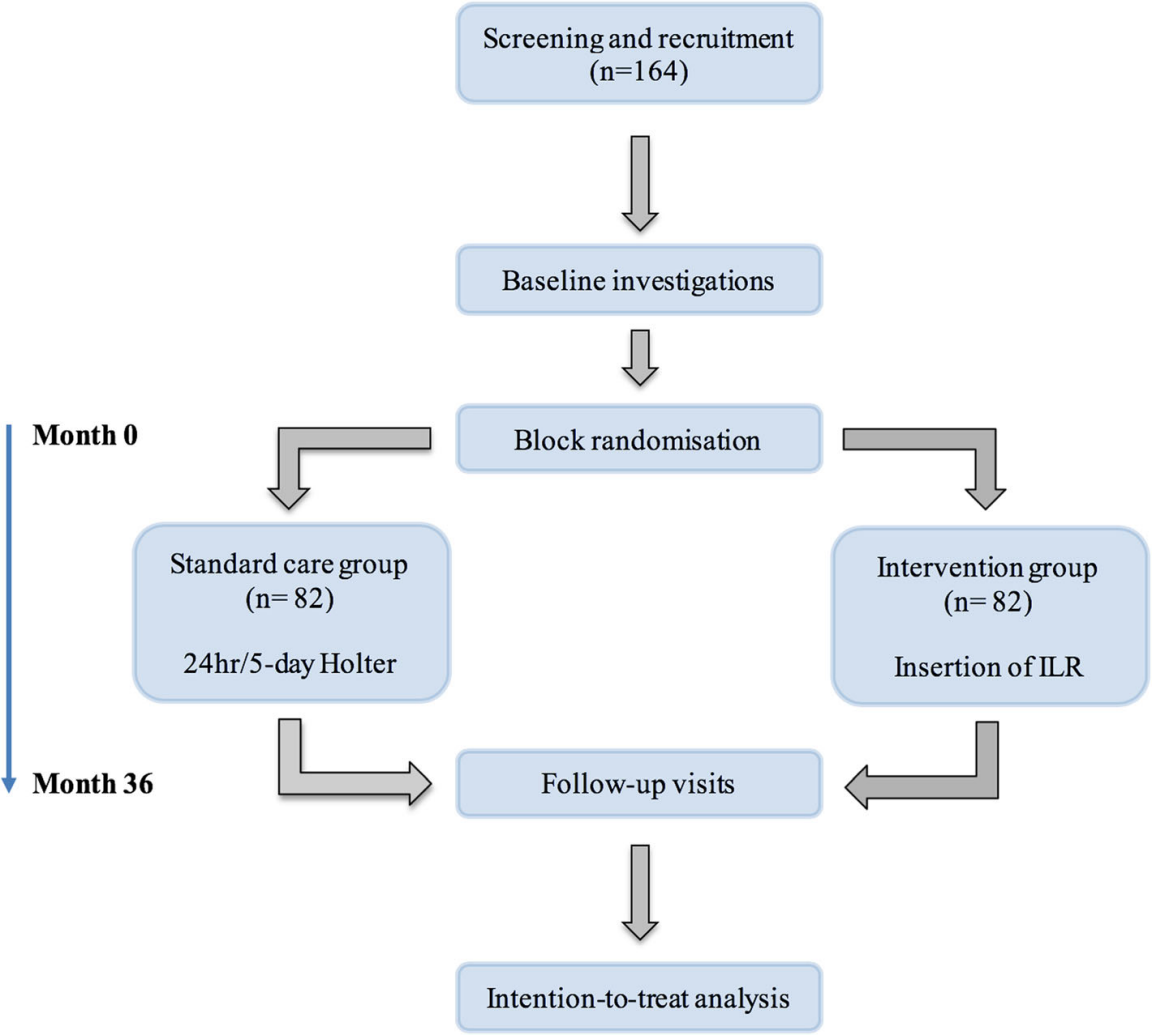
Study timeline. A minimum of 164 participants will undergo baseline investigation and randomisation to standard care with annual Holter monitoring or to intervention with an implantable loop recorder device for continued electrocardiogram (ECG) monitoring. All participants will be followed up at 12 monthly intervals (centre-dependent) for the entire 36-month study period, with investigations repeated at each time-point.

This is a prospective, 5-year, open-label, international, multi-centre randomised controlled trial to evaluate the role of ILR in modifying therapy for arrhythmia in FD. Adults with genetic or enzymatic confirmation of FD who have evidence of cardiac involvement will be recruited from five centres: (1) Queen Elizabeth Hospital, Birmingham, UK; (2) Salford Royal Hospital, Salford, UK; (3) Royal Free Hospital, London, UK; (4) Addenbrookes Hospital, Cambridge, UK; and (5) Westmead Hospital, Sydney, Australia. Participants will be randomly assigned to one of two study arms for cardiac monitoring: (i) standard of care using annual 24-h/5-day ECG monitoring according to local practice or (ii) continuous cardiac monitoring with ILR implantation plus standard of care. A summary of the trial design is shown in Fig. 1.

### Null hypothesis

There will be no difference in the identification of clinically significant arrhythmia that requires treatment modification between patients following standard care alone compared with those following standard care with the addition of ILR monitoring.

### Study aims

This trial will compare the rate of clinically significant arrhythmia requiring intervention identified between the two surveillance modalities. This information will be correlated with cardiac investigations performed at intervals (details described below) and thus the study aims are the following:

1. Identify the true arrhythmic burden in FD.
2. Demonstrate the clinical benefit of ILR in modifying treatment.
3. Provide detailed data regarding specific risk factors predisposing to arrhythmic events.

This study will identify the predictive power of both traditional (LGE, QRS duration, atrial size, and left ventricular (LV) ejection fraction) and novel (troponin, pro-brain natriuretic peptide (pro-BNP), lyso-Gb3, T1 and T2 mapping, and A-ECG analysis) biomarkers. This will provide greater clarity on the risk of sudden cardiac death (SCD) and ultimately guide the formulation of a FD-specific risk stratification tool for primary prevention.

### Participants

Participants will be recruited from Fabry clinics over a 12- to 18-month recruitment period.

Inclusion criteria:

- Adults older than 18 years of age with confirmed FD (genotypically with a homozygous or heterozygous Fabry-specific mutation and/or enzymatically defined by absent or reduced alpha-galactosidase A)
- Evidence of cardiac involvement from FD, including at least one of the following:
  - ◦ Any ECG abnormality associated with FD (Table 1)
  - ◦ Low T1 on CMR imaging (below centre-specific normal range according to sex)
  - ◦ LVH on transthoracic echocardiogram or CMR (defined as maximum wall thickness (MWT) of more than 12 mm)

**Table 1:** Electrocardiogram (ECG) abnormalities associated with Fabry disease

ECG abnormality	
Short or prolonged PR interval (<120 ms or >200 ms)	
QRS duration > 120 ms	
T-wave inversion in at least two contiguous leads	
Prolonged QTc	

Exclusion criteria:

- Patients with an existing cardiac device (PPM, ICD, cardiac resynchronisation therapy (CRT) or ILR)
- Known dual pathology:
  - ◦ Coronary artery disease (positive non-invasive imaging, confirmed myocardial infarction, previous percutaneous or surgical revascularisation). Patients more than 40 years old with symptoms that could be from coronary artery disease will have this excluded.
  - ◦ Cardiomyopathy disease causing mutation (e.g., SCN5 and MYBPC3).

The following features have been linked to an increase in the risk of cardiac arrhythmia and SCD [7]. The study population will be enriched with these arrhythmic risk factors:

- LVH:
  - ◦ Elevated indexed LV mass (greater than two standard deviations above age, sex and body surface area indexed measured on steady-state free precession cine imaging CMR)
  - ◦ MWT of more than 12 mm
- LA dilatation on two-dimensional (2D) transthoracic echocardiography:
  - ◦ M-mode measurement of more than 40 mm or biplane volume of more than 34 mL
- Elevated biomarkers (high-sensitivity troponin T or I above local reference value)
- QRS duration of more than 120 ms on standard ECG
- Presence of LGE on CMR
- MSSI of more than 20 [19].

### Randomisation

**Fig. 2:**
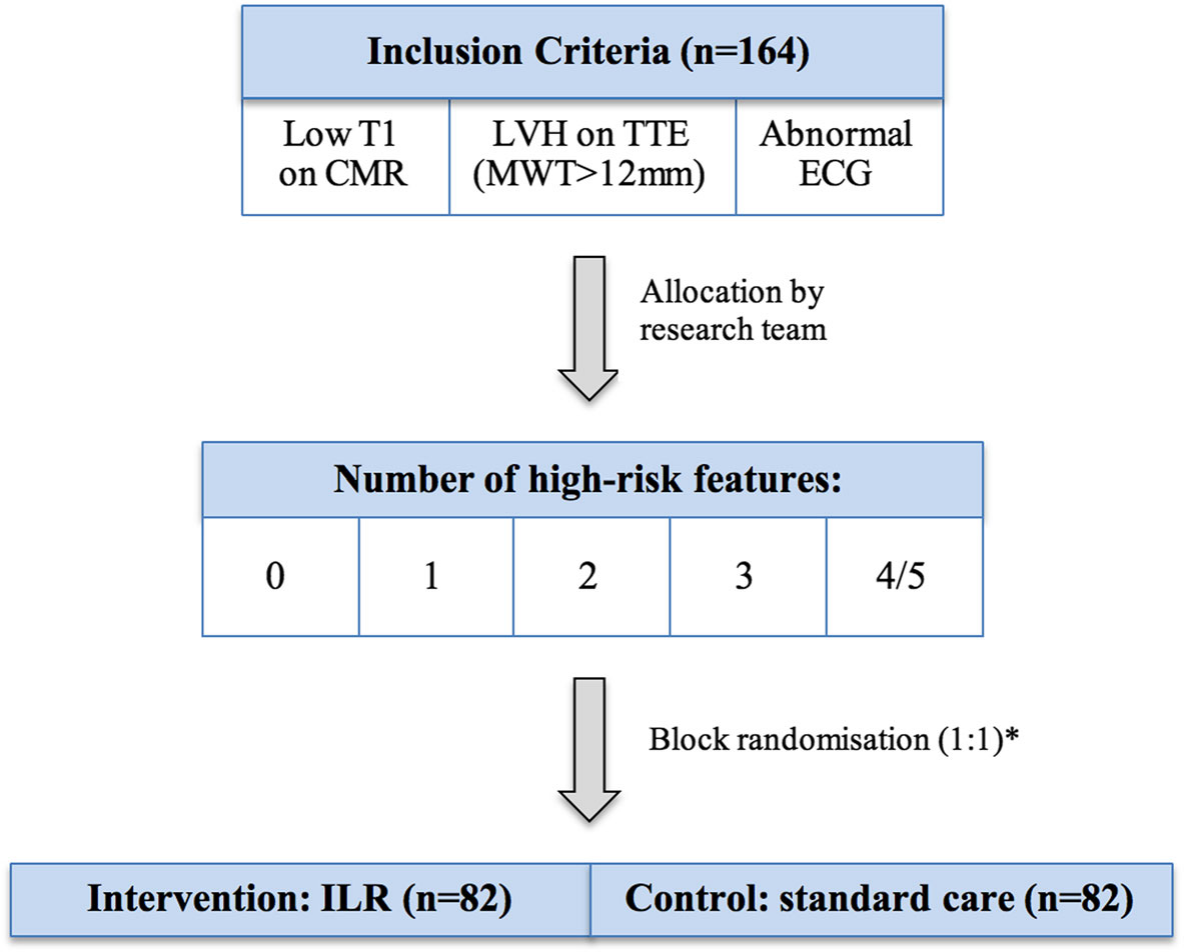
Block randomisation process. The high-risk features include LVH (MWT > 12 mm or elevated LVMi greater than two SD), LA dilatation (M-mode measurement > 40 mm or biplane volume > 34 mL on echo), elevated troponin (above centre specific reference ranges), prolonged QRS duration > 120 ms, presence of LGE on CMR imaging, a MSSI greater than 20. *There will be a variable number of patients from each high-risk feature group to ensure a variable risk profile within the study cohort (zero risk factors – 20 participants, one risk factor – 40 participants, two risk factors – 40 participants, three risk factors – 40 participants, four or five risk factors – 24 participants. *Abbreviations*: *CMR* cardiac magnetic resonance imaging, *ECG* electrocardiogram, *ILR* implantable loop recorder, *LA* left atrium, *LGE* late gadolinium enhancement, *LVH* left ventricular hypertrophy, *LVMi* indexed left ventricular mass, *MSSI* Mainz severity score index; *MWT* maximum wall thickness, *SD* standard deviation, *TTE* transthoracic echocardiography.

A minimum of 164 participants will be recruited and randomly assigned to either the control group receiving standard of care or the intervention group who will have continuous cardiac monitoring with an ILR plus standard of care. A block randomisation process will be performed by an independent statistician in a 1:1 ratio (Fig. 2). Allocation tables will be imported to the Research Electronic Data Capture (REDCap) web application software hosted by the National Hospital for Neurology and Neurosurgery (London). REDCap is a secure web-based electronic software used to facilitate data capture for research studies [20]. All clinical data collected will be entered directly into the REDCap case report forms. Sham devices will not be used in the control group; thus, it will not be possible to blind clinicians, investigators or patients.

### Assessment and follow-up

**Fig. 3:**
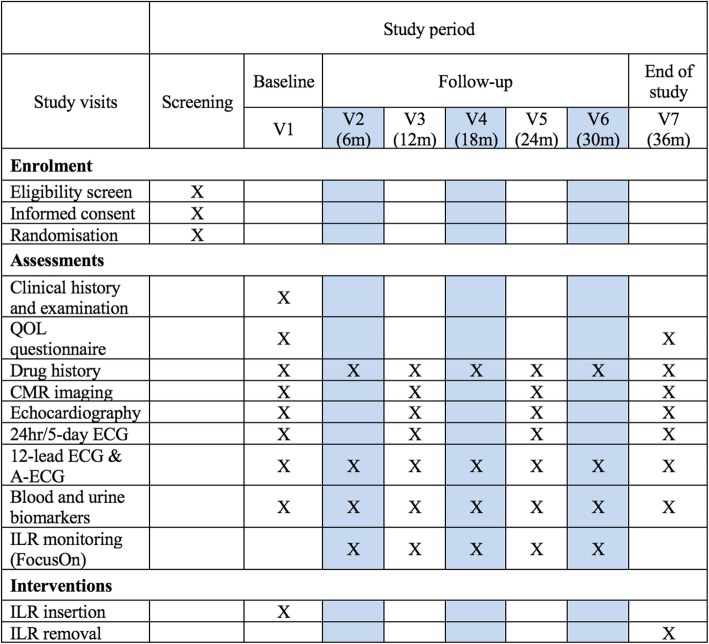
Summary demonstrating participant activity for the duration of the study. All study visits will occur during routine clinical follow-up visits for Fabry disease surveillance, with only two extra hospital visits for screening and implantable loop recorder (ILR) insertion. The shaded columns represent optional monitoring visits that will be centre-dependent. Adapted from SPIRIT (Standard Protocol Items Recommendations for Interventional Trials) figure (2013). *Abbreviations*: *CMR* cardiac magnetic resonance, *ECG* electrocardiogram, *QOL* quality of life.

Following screening assessment for eligibility, all patients will undergo study entry investigations at their baseline visit. Subsequent follow-up will occur for 3 years at 6–12 monthly intervals, although recruiting centres have the option to review patients more frequently depending on clinical need. All study procedures will be repeated at follow-up visits. Figure 3 summarises the follow-up period and clinical procedures that will be undertaken at each time point. The investigations listed below will be carried out on the whole study population:

1. Blood and urine analysis

Routine biochemical and haematological parameters, including plasma lipids, thyroid function, vitamin D level, high-sensitivity troponin T or I (site-dependent) and N-terminal pro brain natriuretic peptide (NT-proBNP), will be analysed. Additional biomarker analysis will include hs-CRP and IL-6. Urine samples will be collected for analysis of albumin-creatinine ratio.

2. Echocardiography

A detailed transthoracic echocardiogram will be performed at each centre by an accredited echocardiographer in accordance with the British Society of Echocardiography Minimum Dataset for a Standard Transthoracic Echocardiogram [21]. Analysis will be performed locally at each centre, and ventricular dimensions, wall thickness, chamber volumes and cardiac output will be evaluated by using standard guidance [21, 22]. Left ventricular diastolic function will be evaluated at rest by using standard guidance. This will include measurement of peak early diastolic (e′) mitral annular velocities at the septal and lateral walls by using pulsed wave tissue Doppler in end-expiration, mitral valve inflow early (E wave) and atrial (A wave) filling velocity, and atrial volumes measured on 2D from the apical four-chamber and modified apical two-chamber views [23]. Global longitudinal strain will be measured by speckle-tracking analysis of 2D images acquired at end-expiration in the apical four-, two- and three-chamber views in accordance with current guidance [24].

3. Cardiovascular magnetic resonance imaging

Participants will undergo CMR imaging as part of the research study or as clinically indicated. These will occur at Birmingham (UK), Barts Heart Centre, Manchester Royal Infirmary, Addenbrookes Hospital and Royal North Shore Hospital (Sydney, Australia). A 1.5-Tesla MR system (Avanto (UK), Aera (Australia), Siemens Healthcare, Erlangen, Germany) and a standard protocol including LV cines in short axis (SAX), four-chamber, two-chamber and three-chamber views (ECG R wave gated steady-state free precession imaging) will be used. Native T1-mapping will be performed before and after contrast administration by using a Modified Look-Locker Inversion (MOLLI) recovery sequence (8-mm slice with a 192 read-out matrix, 6/8 phase partial Fourier with 81% phase resolution, repetition time 2.4 ms, echo time 1.01 ms, 11 phases; 3, 3, 5 scheme). The resulting pixel-by-pixel T1 colour map will be displayed by using a customised 12-bit lookup table, where normal myocardium will be defined as purple, increasing T1 as pink/red, and decreasing T1 as dark blue. T2 colour maps will be similarly formed, and increasing T2 will be shown as orange/yellow. LGE imaging will be performed by using phase-sensitive inversion recovery (bolus administration of gadolinium based contrast 0.1 mmol/kg body weight) to quantify the location, pattern and extent of LGE. Image analysis will be performed offline by an accredited cardiologist or radiologist at each centre by using Cvi42 (Circle Cardiovascular Imaging Inc., version 5.3.4, Calgary, AB, Canada) and will include assessment of ventricular mass, volumes, tissue characterisation with T1/T2 mapping and assessment for the presence of LGE by using methodologies previously described [25].

4. 12-lead ECG and advanced ECG analysis

A standard resting 12-lead ECG (for 10 s at 25 mm/s and 10 mm/mV) will be acquired at annual study visits and stored digitally in .xml format. Abnormal ECGs will be defined as the following: (1) LVH classified by traditional Sokolow–Lyon methodology (SV1 + RV5 or RV6 ≥3.5 mV) and by gender-specific Cornell voltage criteria (RaVL + SV3 ≥2.8 mV in males and ≥2.0 mV in females); (2) resting ST depression or T-wave abnormalities; (3) prolonged QTc (≥440 ms in males and ≥460 ms in females); (4) prolonged QRS duration (≥120 ms) or the presence of complete bundle branch block or both; (5) prolonged or shortened PR interval (>200 ms or <120 ms respectively); and (6) the presence of ventricular ectopy. In addition, a continuous 5-min ECG (1000 mm/s and 10 mm/mV) will be acquired at rest in the supine position by using SpaceEKG technology [26] and stored electronically as an .xml digital file. Subsequent offline analysis will be performed by using A-ECG software (Space EKG Technology, advanced electrocardiogram, version 4.36) (Additional file 1). All patients will undergo annual 24-h or 5-day ECG Holter monitoring and subsequently data will be analysed on Spacelabs software (OSI Systems, Spacelabs Healthcare, Snoqualmie, WA, USA).

The intervention group will undergo additional study procedures, as listed below:

5. Insertion of ILR

ILR devices (Medtronic UK or Medtronic Australia) will be implanted in accordance with centre-specific policies. In the UK centres, the procedure will be performed by the research team using local anaesthetic and will not require overnight hospital admission or continued monitoring by the cardiac physiology department. In Australia, the procedure will occur at Royal North Shore Hospital (Sydney) as a day-case hospital admission and will be performed by a member of the research team who is a senior electrophysiology cardiologist (DW).

6. ILR follow-up

All ILRs for participants within the UK will be followed up using home monitoring (FocusOn, Medtronic and Fysiologic BV, Amsterdam, The Netherlands). This is a continuous cardiac monitoring and triage service. Data will automatically be sent to the research team within the lead study site (Birmingham), and information will be disseminated to the participating clinical sites if required. Surveillance of the Australian participants will be carried out by using remote home monitoring (Medtronic Reveal LINQ Monitoring Service, Medtronic Australia), and data transferred to Royal North Shore Hospital where it will be disseminated to relevant clinical sites if required. Monitoring information will be transferred to the central study site on the basis of suspected clinical urgency (red: urgent, within 24 h; amber: less urgent, within 48 h; green: routine, within weekly reports). Detailed classification of arrhythmia can be seen in Additional file 2.

7. ILR removal

The device will be removed after 3 years of being *in situ* in accordance with centre-specific policy. The ILR device will also be removed in those reaching a study primary endpoint requiring implantation of an alternative cardiac device (PPM, ICD or CRT). These patients will continue ongoing follow-up with arrhythmia surveillance via their new device.

### Outcome measures

#### Primary endpoints

The primary endpoint of this study will include any clinically significant arrhythmia requiring initiation or modification of treatment, which includes the following:

- Atrial fibrillation requiring anticoagulation (defined as an episode of arrhythmia for a duration greater than 30 s) [27]
- Bradyarrhythmia requiring pacing (this would include any symptomatic significant AV block and Mobitz type 2 AV block or complete heart block irrespective of symptoms) [28]
- Supraventricular arrhythmia requiring drug treatment or ablation
- Non-sustained ventricular tachyarrhythmia requiring drug treatment, ICD implantation or ablation (defined as three or more ventricular beats at a rate of more than 120 beats per minute for a duration of less than 30 s) [29, 30].

#### Secondary outcome measures

Secondary outcome measures will be evaluated providing detailed information on arrhythmia in FD. These include the following:

- Quantification of the arrhythmic burden in FD in those with or without LGE
- Characterise the relationship between arrhythmia burden and advanced CMR tissue characterisation techniques (T1 and T2 mapping)
- Assess the value of ECG abnormalities, particularly a QRS duration greater than 120 ms, in predicting arrhythmia
- Assess the predictive value of advanced ECG analysis in arrhythmia
- Evaluate the role of atrial size in burden of AF
- Determine the effect of LVH and LV mass on arrhythmia burden
- Assess the relationship of MSSI (a severity score index used in FD) and burden of arrhythmia
- Evaluate the effect of variations in blood biomarkers (high-sensitivity troponin, lyso-Gb3, hs-CRP and IL-6) on the development of arrhythmia.

All endpoints will be reviewed and verified by a data monitoring committee.

### Statistical analysis

#### Sample size calculation

There is a paucity of knowledge about the burden of arrhythmia in FD; based on the available literature and our own collective experience, the following conclusion can be made about the necessary sample size for this study.

Existing literature has shown that over a 7-year period the incidence of arrhythmia in FD patients under standard follow-up at a specialist centre (with annual ECG recording and symptom driven investigation) is: atrial fibrillation 6%, bradyarrhythmia requiring device implantation 6% and cardiac event-related death 3% [30]. These were evenly spread over the 7-year study period with increased rates in the older age group. Assuming an even spread of events detectable over the 3 years of our study, we estimate the background detection rate of clinically significant events in those meeting the inclusion criteria and following standard care to be 5%.

In an enriched population with advanced cardiac involvement identified by the presence of LGE and LVH, undergoing routine annual 24-h Holter monitoring, the rate of identification of significant arrhythmia and sudden cardiac death was 24.6% [31]. Despite assuming a lower event rate in patients meeting the inclusion criteria for this study, we estimate the detection rate in the study group with ILRs will be 20% due to more accurate identification of all arrhythmia by these devices. With these values, for a given population proportion of 0.05 versus 0.2, sample sizes 82 and 82 in the intervention and control study arms, respectively, and an alpha of 0.05 (two-tailed), the power of the study is 80%. This means that 80% of studies would be expected to yield a significant effect, rejecting the null hypothesis that the two populations have identical event rates requiring intervention.

#### Planned statistical analysis

Statistical analyses will be carried out by using SPSS 23 (IBM Corporation, Armonk, NY). All continuous variables will be expressed as mean ± standard deviation, and all non-continuous data will be expressed as frequencies or percentages. Normality will be evaluated by using the Shapiro–Wilk test. The independent *t* test will be used to compare parametric data, and the Mann–Whitney *U* test will be used for non-parametric data. Chi-squared or Fisher’s exact tests will be used to compare proportions within two independent groups. For comparisons of repeated sample results, repeated measure analysis of variance (ANOVA) with post-hoc Bonferroni correction will be used. Time-to-event (survival) analysis will be performed to evaluate the presence of arrhythmic events during follow-up. Kaplan–Meier curves will be used to estimate the cumulative probability of arrhythmic events, and Cox proportional hazards regression will be used to evaluate the relationship between predefined covariates and outcome events. A *P* value of less than 0.05 will used for statistical significance.

All patients will have a minimum of 18 months’ follow-up for the primary outcome before interim safety analysis, which will be performed on an intention-to-treat basis. The relationship between arrhythmic burden and all other variables will be analysed by using the methods described above. An alpha spending function method will be used for the interim analysis, and a critical *P* value of less than 0.01 will be used to signify statistical significance.

### Additional considerations

#### Adverse events and participant withdrawal

All adverse and serious adverse events (SAEs) will be reported in accordance with standard International Council for Harmonisation Good Clinical Practice reporting guidelines. SAEs will be reported within 24 h of identification to the local study investigator and the chair of the clinical events review committee (CERC). All SAEs will be reviewed for causality and ongoing management requirements. Any serious concerns identified by the CERC may result in modification or termination of the study or participant withdrawal. Complete withdrawal criteria are listed in Table 2.

**Table 2:** Participant withdrawal criteria

Withdrawal criteria	
Participant’s withdrawal of consent	
An adverse event (AE) or serious adverse event (SAE) requiring withdrawal from study (determined by CERC)	
Death	
Substantial protocol deviation	
Investigator or sponsor decision	

## Discussion

The use of ILRs for arrhythmia detection in general cardiology has increased significantly over the last 10 years. Newer-generation devices are now considerably smaller and require a minimally invasive procedure for implantation, thus carrying much lower peri-procedural risk. The diagnostic yield, in general cardiovascular conditions, from ILR devices is much higher than current standard-of-care ECG monitoring (stand-alone 12-lead ECG or ambulatory Holter ECG monitoring) [32]. However, the benefit of ILR in FD is not defined.

FD is a rare genetic condition characterised by multi-organ involvement, and significant morbidity and mortality occur secondary to cardiovascular involvement. Although SCD remains a significant cause of death, the total incidence of malignant ventricular arrhythmia is variably reported in the literature [33, 34], and there is no clear guidance on treatment. The rarity and complexity of this disease process have led to a paucity of large randomised clinical trials in this area, and no studies to date have been able to fully characterise risk predictors of arrhythmia. One small single-centre study [34] demonstrated the extensive arrhythmic burden in progressive disease; however, this included only patients with advanced Fabry cardiac disease and consequently did not fully characterise arrhythmic risk across the spectrum of Fabry cardiomyopathy.

We have also recently shown that the burden of ventricular arrhythmia is much higher than previously thought. In a multi-centre study of 109 FD patients who had previously undergone cardiac device implantation, the incidence of ventricular arrhythmia requiring medical or device therapy was found to be as high as 25.7% over a 5-year follow-up period [35], thus highlighting the extent of arrhythmic burden. Additionally, suspected arrhythmic risk factors, such as LVH, extensive LGE and prolonged QRS duration, were all found to be highly prevalent in this cohort. Detailed risk stratification and direct correlation with arrhythmia across all FD risk subgroups are crucial in developing a greater understanding of those at risk of life-threatening cardiac arrhythmia.

This study will be the first to identify the true arrhythmic burden in FD and demonstrate the clinical benefit of ILR in arrhythmia detection. Additionally, it will characterise arrhythmic risk across a spectrum of patients with FD by using novel biomarker techniques and will allow direct correlation of atrial and ventricular arrhythmia with underlying structural and functional changes.

## Trial status

This study was due to commence recruitment in January 2019 and have a total recruitment period of 18 months.

## Additional files

Additional file 1: Advanced electrocardiogram (ECG) parameters evaluated during study. (DOCX 14 kb)

Additional file 2: Arrhythmia classification guidance for FocusOn home monitoring system. (DOCX 13 kb)

## Data Availability

Data obtained from this study will be disseminated and published through international clinical meetings and peer-reviewed journals. Data will also be presented to local sites and regulatory authorities if required.

## Abbreviations

2D: Two-dimensional
AF: Atrial fibrillation
CERC: Clinical events review committee
CMR: Cardiac magnetic resonance
CRT: Cardiac resynchronisation therapy
ECG: Electrocardiogram
FD: Fabry disease
HCM: Hypertrophic cardiomyopathy
hs-CRP: High-sensitivity C-reactive protein
ICD: Implantable cardiac defibrillator
IL-6: Interleukin-6
ILR: Implantable loop recorder
LA: Left atrial
LGE: Late gadolinium enhancement
LV: Left ventricular
LVH: Left ventricular hypertrophy
Lyso-Gb3: Globotriaosylsphingosine
MSSI: Mainz Severity Score Index
MWT: Maximum wall thickness
PPM: Permanent pacemaker
REDCap: Research Electronic Data Capture
SAE: Serious adverse event
SCD: Sudden cardiac death
