# Supplementary material for: A randomised controlled trial evaluating arrhythmia burden, risk of sudden cardiac death and stroke in patients with Fabry disease: the role of implantable loop recorders (RaILRoAD) compared with current standard practice
Source: Trials. 2019 May 31;20:314. doi: 10.1186/s13063-019-3425-1 (PMC6544923; doi:10.1186/s13063-019-3425-1)
Supplement: Supplementary file 1 — Advanced electrocardiogram (ECG) parameters evaluated during study. (DOCX 14 kb) [file 13063_2019_3425_MOESM1_ESM.docx]

**Additional file 1. Advanced ECG parameters evaluated during study**

| ECG parameter |
| --- |
| Advanced P-wave analysis   - duration - p-wave dispersion - axis - p-wave area   QRS score  Fragmentation of QRS  Spatial QRS-T angle  QT dispersion  T-wave peak to end interval  LVH score |
