# Supplementary material for: A randomised controlled trial evaluating arrhythmia burden, risk of sudden cardiac death and stroke in patients with Fabry disease: the role of implantable loop recorders (RaILRoAD) compared with current standard practice
Source: Trials. 2019 May 31;20:314. doi: 10.1186/s13063-019-3425-1 (PMC6544923; doi:10.1186/s13063-019-3425-1)
Supplement: Supplementary file 2 — Arrhythmia classification guidance for FocusOn home monitoring system. (DOCX 13 kb) [file 13063_2019_3425_MOESM2_ESM.docx]

**Additional file 2. Arrhythmia classification guidance for FocusOn home monitoring system**

| **Red (report immediately)** |
| --- |
| Sustained broad complex tachycardia ≥30secs |
| NSVT >10 beats |
| Pause ≥5secs |
| Bradycardia ≤40bpm (daytime) |
| **Amber (report 24hours)** |
| First episode AF |
| Daily burden AF >1 hour |
| **Green (weekly report)** |
| First atrial tachycardia |
| Daily burden atrial tachycardia >1 hour |
| Presence of AF (not first episode) |
| Symptomatic episode with low priority ECG abnormality |
| False positive (e.g. AF, undersensing, oversensing) |
